# A New Electromagnetic Acoustic Transducer Design for Generating and Receiving S0 Lamb Waves in Ferromagnetic Steel Plate

**DOI:** 10.3390/s17051023

**Published:** 2017-05-04

**Authors:** Jianpeng He, Steve Dixon, Samuel Hill, Ke Xu

**Affiliations:** 1 National Engineering Research Center of Advanced Rolling Technology, University of Science and Technology Beijing, Beijing 100083, China; o832an@hotmail.com; 2 Department of Physics, University of Warwick, Coventry CV4 7AL, UK; S.M.Dixon@warwick.ac.uk (S.D.); samuel.hill@warwick.ac.uk (S.H.); 3 Collaborative Innovation Center of Steel Technology, University of Science and Technology Beijing, Beijing 100083, China

**Keywords:** electromagnetic acoustic transducer, EMAT, NDT, Lamb wave, magnetostriction

## Abstract

Electromagnetic acoustic transducers (EMATs) are non-contact, ultrasonic transducers that are usually kept within 5 mm from the sample surface to obtain a sufficient signal-to-noise ratio (SNR). One important issue associated with operation on a ferromagnetic plate is that the strong attraction force from the magnet can affect measurements and make scanning difficult. This paper investigates a method to generate fundamental, symmetric Lamb waves on a ferromagnetic plate. A coil-only, low-weight, generation EMAT is designed and investigated, operating at lift-offs of over 5 mm. Another design of an EMAT is investigated using a rectangular magnet with a much higher lift-off than the coil, of up to 19 mm. This results in a much lower force between the EMAT and sample, making scanning the EMAT much easier.

## 1. Introduction

An electromagnetic acoustic transducer (EMAT) can generate and detect ultrasonic waves on electrically-conductive samples without making physical contact, making it possible to take measurements on moving or hot objects. Another advantage of EMATs is the capability of generating various ultrasonic wave modes by careful design of the coils and magnets [1,2,3,4,5,6,7,8,9]. However, EMATs have some disadvantages: the signal-to-noise ratio is relatively low compared with piezoelectric transducers and the gap between the bottom of the EMAT and the conductive material is typically limited to less than 5 mm, even after signal averaging and optimized design [10,11].

Traditional EMATs consist of a coil and a magnet, and are used to generate ultrasonic waves in metallic samples that are under test. EMATs generate and detect these ultrasonic waves mainly through two mechanisms: the Lorentz force and magnetoelastic mechanisms [12,13,14,15], depending on the experimental sample properties. The Lorentz force can be produced in any electrically-conducting material. When an alternating current is driven through the coil, it generates a dynamic, time varying magnetic flux density, $B_{d}$ that, in turn, generates an eddy current, $J_{E}$, in the surface region of the metallic plate. The Lorentz force describes the forces generated by the interaction between the eddy current and the magnetic field [16] (both the dynamic magnetic field, $B_{d}$, from the coil and static magnetic field, $B_{s}$, provided by the permanent or electromagnet). This gives rise to a temporally and spatially varying Lorentz force under the EMAT, described by:

(1) $$f=J_{E}\times \left(B_{s}+B_{d}\right)$$

When the dynamic magnetic field is relatively small, there is a linear relationship between the applied static magnetic field and the Lorentz force component of the amplitude of the generated ultrasonic waves. If the applied static magnetic field is parallel to the surface of the sample, then the other main transduction mechanism is typically magnetostriction [12,17]. This occurs only in ferromagnetic media and is highly sensitive to material properties and operational conditions, such as the magnetostriction coefficient λ, relative magnetic permeability μ, and bias magnetic field $B_{s}$. Ferromagnetic materials have a structure that is divided into domains, each of which is a region of uniform magnetic polarization. When a magnetic field is applied, the domains tend to align parallel to the total magnetic field, inducing mechanical strains in the material. Magnetostriction is highly non-linear, and depends on the surface conditions, the previous history of magneto-mechanical loads, and the residual stress [18]. The receiving processes are also different for the Lorentz mechanism and the magnetostriction mechanism. For detection of ultrasonic waves via the Lorentz mechanism, when a passing acoustic wave oscillates the atomic lattice beneath the receiving EMAT, the oscillatory movement in the static field causes an oscillation flow of electrons within the metal plate. The alternating electromagnetic field from the electron flow in the surface of the metal induces an alternating voltage in the coil of the receiving EMAT. The inverse magnetostrictive effect (also known as the Villari effect) is used for the detection of ultrasonic waves via the magnetostrictive mechanism. The time-varying stress induced by the ultrasonic wave, beneath the receiving EMAT, induces a change in the magnetisation of ferromagnetic sample. This results in a time varying magnetic flux density change under the EMAT coil, which induces electric potential difference in the coil. The permanent magnetic field provides a magnetic bias that significantly increases the efficiency of the detection mechanism. A third transduction mechanism on ferromagnetic materials is the magnetization force. When providing a tangential bias magnetic field, a major part of the Lorentz force will be cancelled by magnetization force. The contribution of magnetization force is relatively small compared with the Lorentz force and the magnetostriction force in our EMAT design, so it will be neglected in the subsequent analysis [19].

When designing the EMAT, the important point to bear in mind is that the generation efficiency of the ultrasonic wave is dependent on both the sample under test and the configuration of EMATs. Compared with aluminium, it is generally considered more challenging to perform EMAT measurements on un-oxidised steel because of the lower conductivity and higher density of the steel, both of which serve to reduce the EMAT efficiency in the Lorentz mechanism of operation, whilst the magnetostriction force in ferromagnetic material can also produce forces that contribute to the generation of ultrasonic waves [20,21], especially when the bias magnetic is parallel to the sample surface.

In this paper, we designed a coil-only EMAT, which generates Lamb waves in mild steel plate via the interaction between the steel plate and the dynamic magnetic field generated by the coil. Our experimental results demonstrated that the transduction efficiency in the steel plate is higher than that in aluminium plate due to the ferromagnetic nature of the steel and the S0 mode is mainly produced by magnetostrictive mechanism. A rectangular magnet is then applied to provide a bias magnetic field for the coil-only EMAT to enable it to detect ultrasonic signals and also to increase the efficiency of the ultrasonic generation. The results show that the EMATs are able to work over a wide range of lift-offs and the attraction force between the EMAT and the steel plate are reduced dramatically.

## 2. Lamb Waves

Lamb waves are of practical importance in guided-wave inspection and have been widely utilised in modern engineering for crack detection [22], texture measurement [23], and corrosion monitoring [24]. Lamb waves are capable of propagating over relatively long distances with low attenuation and large areas can be inspected efficiently. However, the multimode and the dispersive nature of Lamb waves can make the interpretation of the received signal difficult. In order to simplify the analysis of the signal in our experiments, the current drive to the EMAT coil is at a sufficiently low frequency to excite only the fundamental modes with any significant amplitude. Whilst the A0 mode may have better detection resolution to defect characterisations because of the shorter wavelength compared with S0 mode at the same frequency, the A0 mode is also highly dispersive at low frequencies. This dispersion has been exploited to measure sample thickness, but in some cases the dispersive nature of the A0 mode can make defect detection more difficult. In contrast, the fundamental symmetric or S0 Lamb wave is less dispersive at low frequencies, making the analysis of the signal simpler, to some extent.

## 3. Design of a Coil-Only EMAT

### 3.1. Theoretical Analysis

**Figure 1:**
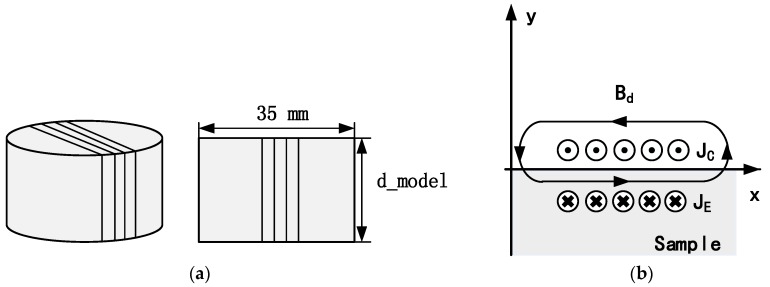
(**a**) Schematic diagram of the EMAT construction. The gap between the coils is depicted much larger than the actual EMAT for clarity. (**b**) The cross-sectional view of the eddy current and dynamic magnetic field generated by linear coils.

Linear coils have been used widely to generate both Lamb and Rayleigh waves for a range of applications [25,26]. As shown in Figure 1a, the conventional linear coil can be constructed by wrapping a single layer of insulated (lacquered) copper wire around a cylindrical magnet. EMATs with this configuration are difficult to operate at a lift-off distance over 5 mm because both the magnetic flux density and the sensitivity of the coil to generate or detect eddy currents in the sample surface decrease rapidly with an increase in lift-off distance. The coil, wound around the sides and top of the permanent magnet, can also dissipate energy as eddy currents can also be generated in the magnet, leading to the reduction of EMAT efficiency. Additionally, the magnet attracts ferromagnetic particles, which may cause mechanical damage to the EMAT and the test object. Previous studies have shown that it is possible to achieve ultrasonic generation with the ‘self-field’ generated by the coil [16]. The contribution of the dynamic field should not be ignored because the displacement due to the dynamic magnetic field may exceed the displacement caused by the static magnetic field when the excitation current is large, such as in the order of hundreds of amps [27]. Compared with the meander coil in [27], a linear coil is more feasible for generating high-intensity dynamic magnetic fields, because the dynamic magnetic field produced by each turn of the coil adds constructively. Compared with aluminium, it is much more practicable to generate a strong magnetic field in steel because of the ferromagnetic nature of the steel.

**Figure 2:**
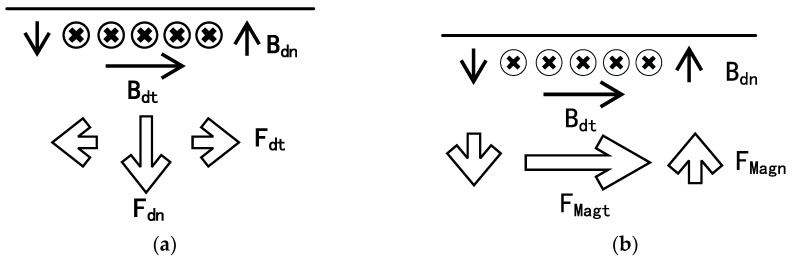
Forces that produced by the coil-only EMAT. $B_{dt}$ and $B_{dn}$ are transverse and normal component of dynamic magnetic field. (**a**) Lorentz forces that arise due to the interaction of the dynamic magnetic with the eddy current. (**b**) Magnetostriction forces that arise due to magnetostrictive mechanism.

When designing a coil-only EMAT, the magnet in Figure 1a is substituted by a 3D-printed, cylindrical plastic model. The diameter is 35 mm, whilst the d_model parameter represents the height of the model. Figure 1b shows the eddy current and dynamic magnetic field produced in the test piece. The coordinate reference frame for the model is placed on the surface of the plate. The x-axis coincides with the surface of the plate. The positive part of y-axis refers to the air domain and the negative part of the y-axis represents the metallic plate. The coil provides a dynamic magnetic field parallel to the surface of the plate. Figure 2a shows the forces that arise due to the interaction of the dynamic magnetic field with the eddy current, which is based on the Lorentz mechanism. Figure 2b depicts the forces generated by magnetostriction. For the Lorentz force, the out-of-plane component is large, while the in-plane component is relatively small. On the contrary, magnetostriction mainly produces in-plane force and the out-of-plane component is relatively small. In the low frequency-thickness regime, the vibration associated with S0 mode is predominantly in-plane [15], meaning that the S0 Lamb wave mode can be efficiently generated by the in-plane, magnetostrictive forces. The A0 mode is mainly generated by the out-of-plane force, which is through the Lorentz mechanism in our design.

### 3.2. Simulation of Magnetic Flux Density with Lift-Off

**Figure 3:**
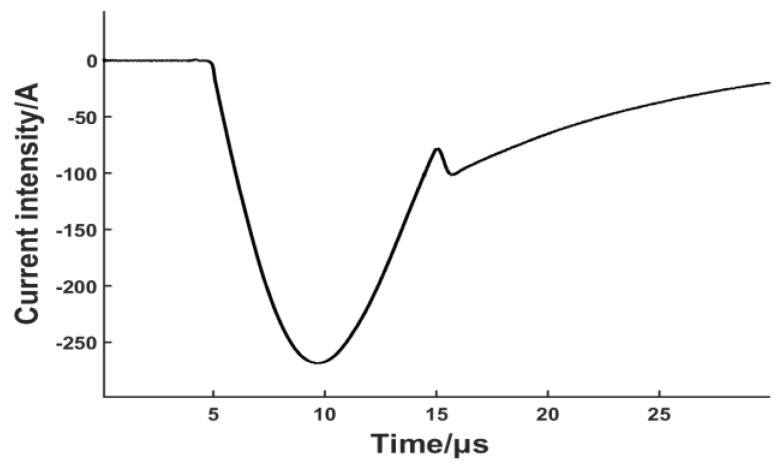
Experimental excitation pulse.

When considering how lift-off will change the coil-only EMAT performance, the dynamic magnetic field generated by the coil is expected to be the most important factor. A finite element (FE) numerical model was firstly implemented in Comsol Multiphysics (5.1 version, COMSOL Inc. Burlington, MA, USA) to simulate the dynamic magnetic field density in the skin depth of the metallic plate. The coils used in the FE model employed a single layer of 10 turns of 0.68 mm diameter wire. The current pulse used in our simulation is broadband pulse with frequency component mainly from 0 to 300 kHz. Low frequency ensures that the wavelength of the Lamb wave is much larger than the sheet thickness, so higher-order wave modes can be suppressed. The pulse through the coil is shown in Figure 3 and the data was collected by measuring the voltage across a resistor in series with the coil-only EMAT. The current peak value is approximately 270 A with several microseconds duration.

The lift-off distance started from 0 mm and increased to 14 mm in 2 mm increments. The simulation was done on both steel and aluminium plate in order to compare the difference of magnetic flux density due to the ferromagnetic properties. The x component of the magnetic flux density (parallel to the plate surface), at the same position in the skin depth, was collected.

**Figure 4:**
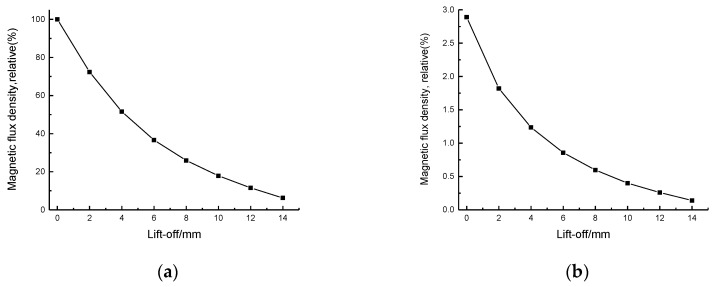
The variation of x component of magnetic flux density as the lift-off of coil-only EMAT is varied on steel (**a**) and aluminium (**b**) samples. The results are normalized to aid comparison, with the magnetic flux density at a 0 mm lift-off defined as 100%.

The results are depicted in Figure 4a,b. The magnetic flux density generated by the dynamic field in steel and aluminium plate both decrease exponentially. If the x component of magnetic flux density in steel at a lift-off of 0 mm is defined as 100%, the value at the lift-off of 0 mm on aluminium is only 2.89%. The magnetic flux density in the steel is obviously stronger than that in aluminium at all lift-off distances because of the higher relative permeability. When the coils are placed on a ferromagnetic plate with high magnetic permeability, most of the magnetic flux is confined in the plate and parallel to the surface inside the plate. Although the magnetic flux density in the steel plate drops dramatically at low lift-offs, it is still possible to generate a strong horizontal dynamic magnetic field in the surface region of the steel plate. The y component of the magnetic flux density was also collected and the field intensity is insignificant compared with the x component.

### 3.3. Experimental Procedure

EMATs were set in a ‘pitch-catch’ arrangement, with one EMAT generating a signal that is received by a second one a short distance away. Both a non-oxidized mild steel plate and an aluminium plate were used in the experiment, with a thickness of 1 mm in both cases. The generation EMAT was constructed by wrapping a single layer of insulated copper wire around the 35 mm diameter 3D-printed plastic model, which has a height equal to 20 mm (d_model = 20 mm in Figure 1a). Both the diameter of the coil and number of turns were varied in order to find the optimum conditions for the maximum peak-to-peak amplitude in the steel plate, and it was found that the optimized coil consisted of 10 turns of 0.68 mm diameter insulated wire. The receiving EMAT employed a single layer of 40 turns of 0.1 diameter wire wound onto a cylindrical permanent magnet (35 mm in diameter and 20 mm in height), which provided a static magnetic field normal to the plane of the steel plate. The receiving EMAT is predominantly sensitive to in-plane vibration, but will have some out-of-plane motion sensitivity because of the fringing field of the permanent magnet. The lift-off of receiver was kept constantly at 0 mm during the whole experimental process.

**Figure 5:**
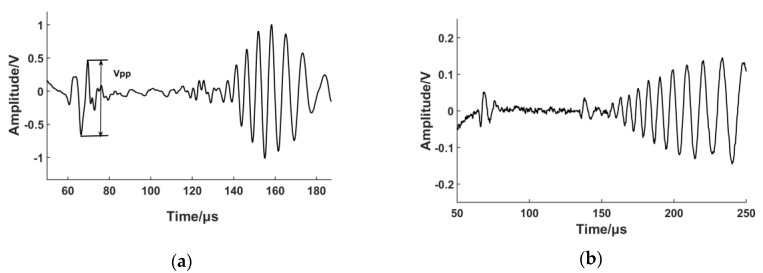
Pulse-echo signal from the coil-only EMAT on steel (**a**) and aluminium (**b**) samples. The amplitude of S0 and A0 mode is much larger in the steel sample.

A low-pass filter, with a cut-off frequency of 500 kHz, was used in order to reduce the level of noise. The received signal was then transmitted to a broadband pre-amplifier with a gain of 50 dB. The pre-amplifier was connected to a digital oscilloscope with 8-bit resolution to record signals in the time-domain, which were averaged 32 times before storage. The results are shown in Figure 5. The group velocity of S0 mode was calculated and the result corresponds with theoretical calculation. The S0 mode shows less dispersion and is temporally sharp due to its broadband nature. V_pp_ shown in Figure 5 is the peak-to-peak value of the S0 Lamb wave. A larger V_pp_ value is often considered a higher transduction efficiency in EMAT design and this value will be used to evaluate the EMAT performance in our experiments.

**Figure 6:**
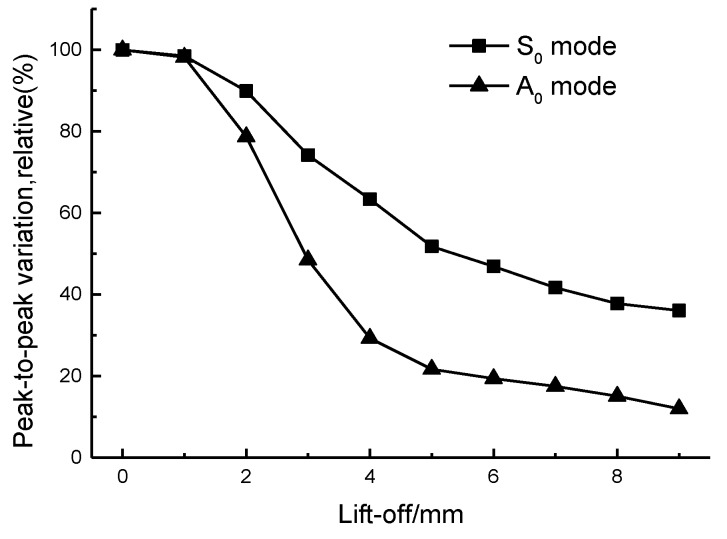
The variation in the peak-to-peak amplitude of S0 and A0 mode. The results are normalized with the peak-to-peak amplitude at a 0 mm lift-off defined as 100%.

The experimental results illustrate that coil-only EMAT driven by large current could generate fundamental Lamb waves in ferromagnetic plate more efficiently than in aluminium plate. The peak-to-peak amplitudes of S0 Lamb waves are 1.1 V and 0.1 V, respectively. Based on the theoretical analysis in Section 3.1 and simulation results in Section 3.2, the increased amplitude of the S0 mode is caused by the magnetostrictive force on the ferromagnetic plate, whilst the increase in the A0 mode amplitude is due to the enhancement of the x-component of the magnetic flux density. In order to prove this hypothesis, an experimental test of transducer lift-off was carried out. The lift-off distance was increased using plastic spacer in a 1 mm step normal to the steel plate and measurements were taken after each step, up to a maximal lift-off of 9 mm. The thickness of the plastic spacer was measured by a micrometer and the maximal relative error was 1.7%. Figure 6 illustrates how the peak-to-peak amplitude of S0 and A0 mode varies with the increment of the lift-off distance.

**Figure 7:**
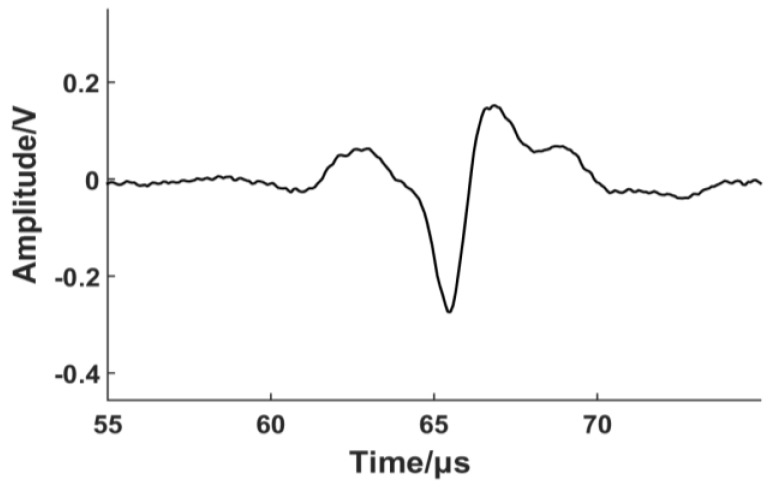
An averaged A-scan of S0 mode Lamb wave when the lift-off distance is 10 mm.

We can observe that the amplitude of the A0 mode attenuates faster than that of the S0 mode. The main reason is that, with the growth of lift-off distance, the Lorentz force falls off more rapidly as both the image current and dynamic magnetic field decrease exponentially. The force caused by magnetostriction simply varies with the decrease of dynamic magnetic field, so the Lorentz force decreases more rapidly than the magnetostriction force with the rise of the gap between EMAT and the ferromagnetic plate. In the low lift-off range (lower than 1 mm), the amplitude varies very little because both the Lorentz force and the magnetostriction force contribute to the generation of ultrasonic waves. A portion of the force produced due to magnetostriction may be counteracted by the force generated by the Lorentz force. Additionally, the magnetostrictive effect is non-linear, making the analysis of the signal complicated. When the lift-off distance is higher than 2 mm, the main transduction mechanism for S0 and A0 become the magnetostriction force and Lorentz force, respectively, and the peak-to-peak values decrease almost exponentially. The amplitude is relatively stable after 7 mm, but the peak-to-peak amplitude is smaller. Figure 7 depicts the A-scan of the S0 Lamb wave generated by the coil-only EMAT in our experiment when the lift-off distance is 10 mm. The result demonstrates that it is possible to generate an S0 Lamb wave with a high signal-to-noise ratio at higher lift-off distance via magnetostriction because eddy current generation is not a requisite factor for the magnetostrictive effect.

It is important to note that the top layer of the linear coil shown in Figure 1 can also produce a dynamic field in the steel plate in the opposite direction to that from the bottom layer, so we need to ensure the dynamic magnet field from the top layer does not reduce the magnetic field intensity in the surface region of the steel plate. If the maximum operational lift-off distance is D_max_, the following equation should be satisfied:

(2) $$d\_\mathrm{model}+D\geq D_{\max}$$

In Equation (2), d_model is the height of the plastic model as shown in Figure 1. D represents the operational lift-off distance of the EMAT, which is the distance between the bottom layer of the coil and the steel plate. (d_model + D) is the lift-off distance of the top layer coil. This equation can ensure that the steel plate is not influenced by the dynamic magnetic field produced by the top layer coil.

Previously-published work has shown that the angle and magnitude of the bias magnetic field has important influence on magnetostrictive transducer efficiency [28,29]. A block NdFeB magnet was employed for the purpose of investigating the influence of the bias magnetic field to the excitation efficiency in the following section.

## 4. Generation and Reception of S0 Lamb Wave Based on the Magnetostrictive Mechanism

### 4.1. Design of the Magnetostrictive EMAT

**Figure 8:**
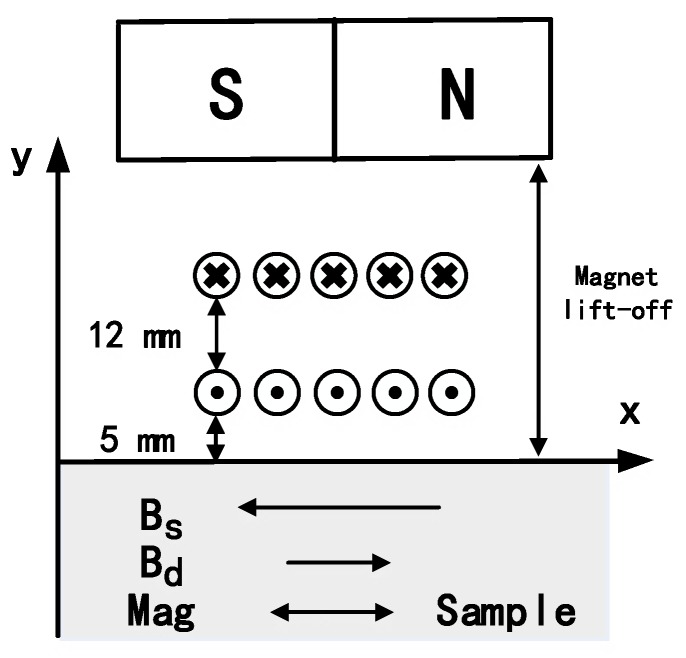
Magnetostrictive EMAT based on the coil-only EMAT described in Section 3. The operational lift-off distance of the coil is 5 mm, so the influence of the Lorentz force can be neglected. The height of the plastic model is set equal to 12 mm (d_model = 12 mm in Figure 1) to avoid interference from the top layer.

The magnetostrictive EMAT (shown in Figure 8) consists of a magnet and a coil-only EMAT described in Section 3. The magnet used in the experiment is a block NdFeB magnet (N40 grade with a residual magnetization of 1.26 T) with a nickel (Ni-Cu-Ni) coating and it is polarised, such that the magnetic axis is parallel to the steel plate.

**Figure 9:**
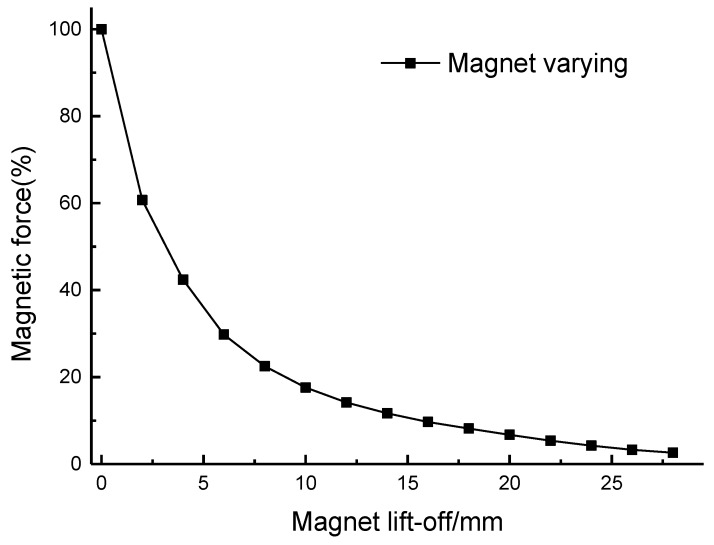
The variation of magnetic force between the magnet and steel plate as the magnet lift-off distance is varied.

This configuration is able to reduce energy dissipation because less eddy currents can be generated in the magnet. Another advantage is that it can reduce the magnetic attraction force between the magnet and the ferromagnetic particles. A finite element method (FEM) model was constructed using COMSOL, consisting of a rectangular magnet and steel plate. After defining the geometry corresponding to the magnet arrangement and ferromagnetic plate, a residual magnetization of 1.26 T was applied to the model along the *x*-axis. All other magnetization components were set to zero. Figure 9 shows the magnetic force as a function of lift-off distance and the force drops dramatically at low lift-off distance. This reduced magnetic attraction can diminish the interference with signal measurements and decrease the risk of mechanical collision during the testing process.

**Figure 10:**
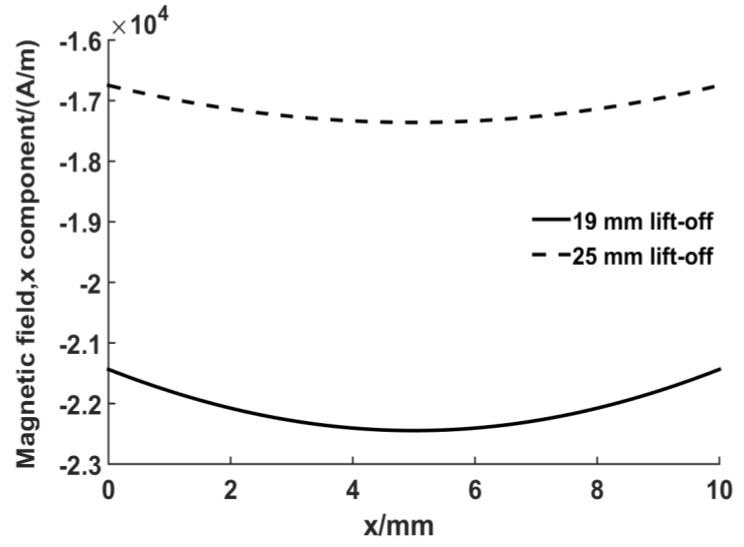
The x component of the magnetic field at the surface when the magnet lift-off is 19 mm and 25 mm.

The minimum lift-off distance of the magnet is 19 mm due to the present of the plastic model and coils in Figure 8. At this lift-off distance, the magnetic force has dropped to 10% of the maximum value. The magnetic field strength was also calculated using the FE model, with Figure 10 depicting the simulation result at the surface of the steel plate when the magnet lift-off is 19 mm and 25 mm. The static magnetic field provided by the magnet is about 22 kA/m and 17 kA/m respectively. The magnetostrictive strain coefficients are related to the slope of the magnetostriction curve and the slope value in mild steel is large when the bias magnetic field $\bar{H}$ is located in a certain interval [29]:

(3) $$16\;\mathrm{kA}/m\leq \;\bar{H}\leq 32\;\mathrm{kA}/m$$

In this interval, greater dynamic strain can be produced, which leads to a larger ultrasonic signal strength.

An experiment was conducted to study the influence of a bias magnet on the excitation efficiency. In order to reduce influence from Lorentz force and design EMATs based on the magnetostriction mechanism, the lift-off of the coil was kept at 5 mm and the lift-off of the magnet varied. The impact from the top layer coil could be neglected at the lift-off values higher than 19 mm.

**Figure 11:**
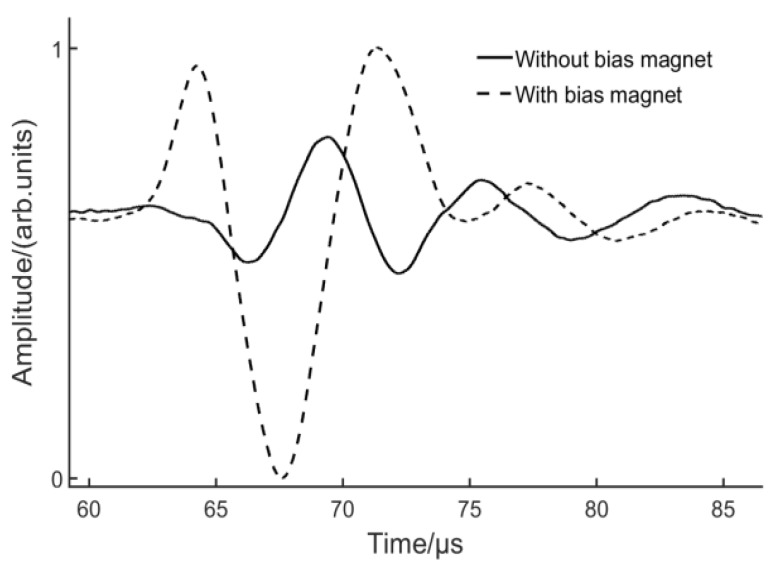
S0 Lamb wave generated by the EMATs with/without a bias magnet.

**Figure 12:**
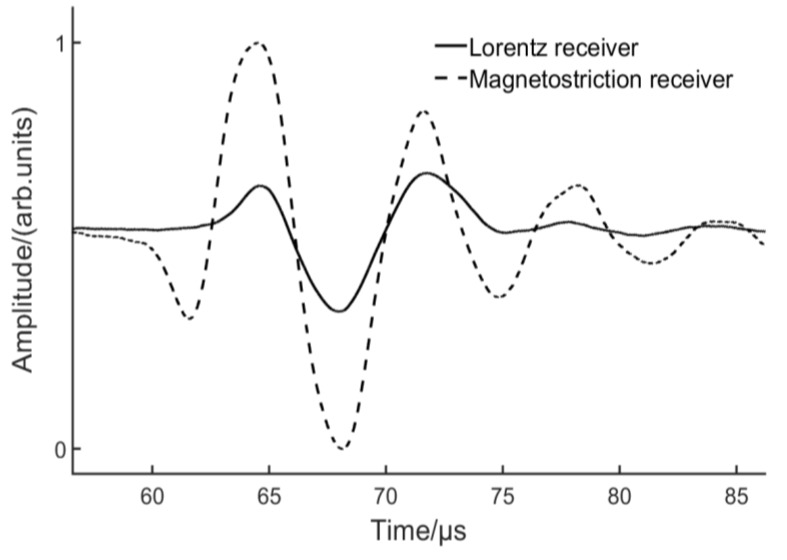
S0 Lamb wave received by the EMATs based on Lorentz/magnetostrictive mechanism.

Figure 11 depicts the comparison between the coil-only EMAT and EMAT with a bias magnet. The lift-off of the Lorentz receiver was kept at 0 mm in order to capture more energy. With a bias magnetic field, the wave packet broadens and the peak-to-peak value triples. For the coil-only EMAT, magnetization can be associated with domain wall movement induced by the dynamic magnetic field. When a bias magnet is utilized, magnetization is expected to change primarily by reversible domain rotations [30]. The experimental result demonstrates that the new configuration is able to generate the S0 Lamb wave more efficiently. This raises the question of whether the same configuration can be used as a receiver. A receiving EMAT was also manufactured by winding a single layer of 20 turns of 0.1 diameter wire, as shown in Figure 8. An experiment was carried out to compare the receiving capability of the EMATs based on Lorentz mechanism and magnetostrictive mechanism. The Lorentz receiver has been introduced in Section 3.2 and the lift-off was set to 0 mm. For the magnetostrictive receiver, the lift-off of the coil and magnet were 5 mm and 19 mm respectively. Figure 12 depicts that the peak-to-peak value from the magnetostrictive receiver was two-fold higher than that from the Lorentz receiver. Furthermore, more peaks can be observed using the magnetostrictive receiver.

### 4.2. Experimental Study of Magnetostrictive EMAT Lift-Off

**Figure 13:**
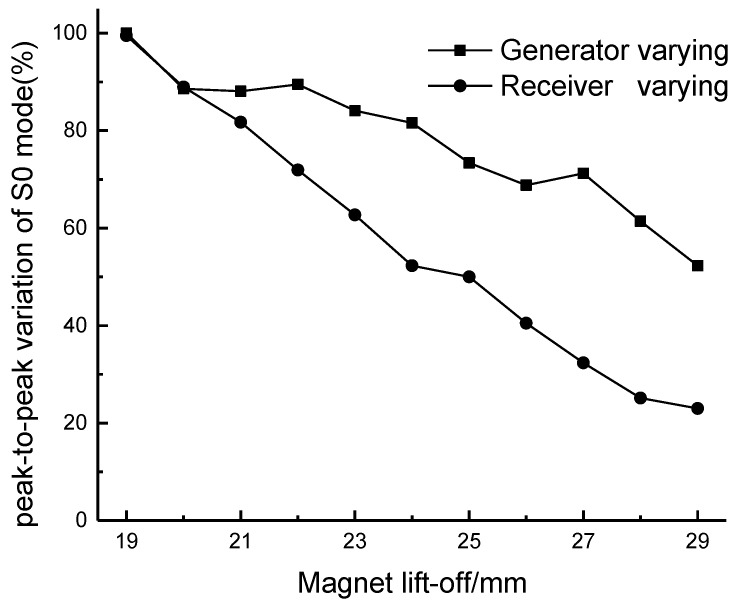
The variation in the peak-to-peak amplitude of the S0 Lamb wave. The lift-off of the coils was kept at 5 mm. The magnet of one EMAT is varied with the other transducer at a fixed magnet lift-off.

Obtaining experimental lift-off data on a steel sample is easier in our experiment compared with traditional EMATs because the steel sample could not be lifted by the magnetic attraction. In the experiment, the lift-off distances were controlled by plastic spacers with different thicknesses. The lift-off of the coils was kept at 5 mm consistently, to provide a sufficient dynamic magnetic field and the lift-off of the magnet varied. Two procedures were performed: the magnet lift-off of the generator was maintained at 19 mm and the magnet of the receiver varied, and then the magnet of the receiver was fixed at 19 mm and the magnet of the generator varied. The variable magnet lift-off started at 19 mm and increased to 29 mm in 1 mm increments. The peak-to-peak value of each step was recorded. Figure 13 reveals the relative value of the amplitude as a function of magnet lift-off distance.

The generator output varies slower with changes in the magnet lift-off. For the receiver, the signal attenuates almost linearly. It could be expected that signals could be generated and received at a magnet lift-off of 25 mm. The newly-designed EMATs were tested on different kinds of ferromagnetic materials. The results show that the EMATs can be successfully utilized to inspect various types of ferromagnetic steel plate. However, the magnetostriction curve is dissimilar for different ferromagnetic materials and the results depend significantly on the material properties. The lift-offs of the coil and magnet should be optimized for each kind of ferromagnetic steel sample.

## 5. Conclusions

Electromagnetic acoustic transducers are one of the most important non-contact methods for defect detection and material characterization. However, traditional linear EMATs, which are usually constructed by wrapping a signal layer of insulated copper wire around a magnet, need to be kept close to the sample surface to obtain a sufficient SNR. When conducting measurements on ferromagnetic samples, things become more complicated because the attraction between the magnet and the sample may disturb the measurements, or even lead to mechanical damage to the transducers. A coil-only EMAT was firstly designed and investigated. The amplitude of both S0 mode and A0 mode are larger in mild steel than in aluminium. The Lorentz force from the interaction of the dynamic magnetic field and eddy current mainly causes out-of-plane force. The enhancement of the magnetic field in mild steel is the primary reason for the A0 mode increment. The magnetostriction force is the main source of the in-plane force, which produce the S0 Lamb wave efficiently. The coil-only EMAT is able to operate over a larger range of lift-offs than conventional EMATs because eddy current generation is not a requisite factor for the magnetostrictive mechanism. Based on the coil-only EMAT, a horizontal bias magnetic field was applied to increase the generating and receiving efficiency. This arrangement is able to generate and receive S0 Lamb waves predominantly via the magnetostrictive mechanism and the generating and receiving efficiency both increased by a factor of about three. The block magnet can provide a bias magnetic field over a 19 mm lift-off distance with the coil fixed at a lift-off of 5 mm, leading to a dramatic decrease of the magnetic force between the magnet and ferromagnetic samples. Compared with traditional EMATs, the higher operational lift-off distance and smaller magnetic force make the new EMAT more suitable for commercial environments.
